# Running Cadence and the Influence on Frontal Plane Knee Deviations

**DOI:** 10.3390/clinpract14060195

**Published:** 2024-11-14

**Authors:** Jacob R. Peterson, Collin R. Sanders, Nathan S. Reynolds, Conner A. Alford, Michael J. Platt, Jeffrey J. Parr, Felix Twum, James R. Burns, David R. Dolbow

**Affiliations:** 1 Physical Therapy Department, William Carey University, Hattiesburg, MS 39401, USArburns@wmcarey.edu (J.R.B.); ddolbow@wmcarey.edu (D.R.D.); 2 School of Health Professions, University of Southern Mississippi, Hattiesburg, MS 39406, USA; felix.twum@usm.edu; 3 College of Osteopathic Medicine, William Carey University, Hattiesburg, MS 39401, USA

**Keywords:** running, cadence, knee, injury prevention, patellofemoral pain

## Abstract

**Background:** Patellofemoral pain is one of the most common injuries in recreational runners, with significant implications for dynamic knee valgus. The knee valgus angle can be corrected surgically or with a more conservative non-operative approach. Increasing running cadence may be an effective biomechanical gait retraining intervention to reduce knee valgus and thus patellofemoral pain. The primary purpose of this study was to examine if an increase in cadence could change the knee valgus angle. **Methods:** Ten asymptomatic recreational runners were recorded running on a treadmill during control and experimental intervals. Each interval lasted five minutes, and participants ran at 100% and 110% of their baseline cadence. Peak angles of knee valgus were compared between both intervals using the video analysis software application Dartfish Express. A paired sample, a two-tailed *t*-test, was used to determine the significant difference between bilateral frontal plane knee angle measurements during both intervals. **Results:** The average decrease in knee valgus measured in control versus experimental intervals was 2.23° for the right leg and 2.05° for the left leg, with a significance of *p* < 0.001 and *p* < 0.001, respectively. **Conclusion:** The results indicated a statistically significant decrease in angles of dynamic knee valgus, attributable to increased cadence. These changes in knee valgus angle are likely to have a positive impact on preventing and reducing pain associated with PFP.

## 1. Introduction

Patellofemoral pain (PFP) or anterior knee pain, more colloquially known as runner’s knee, is one of the most common overuse injuries seen in recreational runners [1]. Key symptoms typically include insidious pain in the anterior portion of the knee, localized swelling, worsening pain with activity, and as the condition progresses, grinding of the patella and a feeling of giving way [2]. PFP has a reported annual incidence of about 23% in the general population [3], with a slightly higher incidence in athletic populations [4]. It has also been well documented that females have an increased incidence compared to their male counterparts. The relevance of PFP is emphasized by the fact that 70% to 90% of individuals may continue to face recurrent or chronic symptoms [5,6,7]. Although the proposed etiology of PFP may be multifactorial and definitive causal factors remain ambiguous, evidence from the current clinical practice guidelines suggests that resultant hip weakness, specifically in the external rotators, post-PFP development contributes to poor lower extremity biomechanics in regards to patella tracking [2]. Individuals diagnosed with PFP consistently exhibit decreased strength in hip abductors and external rotators compared to controls [8,9]. Additionally, the literature suggests there is a moderate negative correlation between the strength of the hip abductors and external rotators and the valgus motion of the knee [9,10].

Knee valgus, characterized by the inward displacement of the knee joint relative to the hip and ankle, can be addressed through surgical and non-operative interventions. While surgical approaches, such as osteotomies or total knee arthroplasty, aim to correct structural misalignments through invasive procedures, non-operative management focuses on dynamic valgus, the functional malalignment that occurs during movement. It is crucial to understand that unlike surgical interventions, which provide more immediate structural changes, non-operative management of dynamic valgus requires consistent effort and adaptation. The effectiveness of such interventions, including gait retraining, depends on continuous practice and reinforcement to achieve lasting improvements in knee biomechanics.

While the knee typically has a structural valgus angle, this angle is increased during the stance phase of walking and running. Previous studies found that individuals diagnosed with PFP were characterized by increased knee valgus during single-limb stance [11,12]. Increased knee valgus angles are directly related to increased contact pressure between the lateral facet of the patella and the lateral femoral condyle, with implications of patellofemoral cartilage degradation and pain over time due to poor patella tracking [11]. It should also be noted that dynamic patellar malalignment combined with increased dynamic knee valgus was explicitly observed in participants with PFP and attributed to poor biomechanics rather than a structural fault [9]. To affect the amount of valgus at the knee, biomechanical changes, proximally rather than distally along the kinetic chain, have been proposed [13,14]. Strengthening of the hip abductors and external rotators to reduce dynamic knee valgus and correct lower extremity biomechanics in symptomatic individuals has been suggested as a plausible solution, with alterations in running cadence showing promise in mediating insufficient gluteal strength and resultant knee valgus [14].

Increased cadence is interpreted as an increase in the step rate over a fixed distance and necessitates a proportional decrease in step length and increased step frequency when running at the same speed [15]. Research shows that increasing cadence can reduce running pain and improve knee function [16]. Improved biomechanics (i.e., reduced dynamic knee valgus and greater relative hip strength) along the kinetic chain have been observed with a cadence increase as low as 5% and, more consistently, as high as 10% [15,17,18,19,20]. More importantly, a decrease in the peak forces required of the gluteal muscles has been observed with a 10% increase in running cadence due to a reduced abduction moment experienced at the hip [19]. It appears there is an inverse relationship between cadence and peak ground reaction forces, with reduced forces experienced along the kinetic chain [19,21]. Specifically, at the knee, participants experienced lower peak patellofemoral and tibiofemoral forces during increased cadences, along with reduced patellar tendon forces [15,19,22,23]. The clinical practice guidelines on patellofemoral pain state that increasing cadence may be used to reduce pain [2].

Current literature highlights a relationship between biomechanical alterations at the hip, contributing to dynamic knee valgus. An increased running cadence may reduce forces throughout the lower extremity during stance, particularly at the hip. However, current literature analyzing the direct impacts of cadence and changes in the amount of dynamic knee valgus is limited. Previous research has identified that increasing cadence can reduce pain levels and improve knee function in patients with PFP [11], however, it has not been determined what mechanical factors aid this positive change. The primary purpose of this study was to examine whether an increase in cadence could change the knee valgus angle. It is hypothesized that with an increased cadence from an established baseline, participants will exhibit a decrease in measures of dynamic knee valgus. If a relationship can be established, this research will contribute to the current literature and hopefully serve as a proof of concept for improving biomechanics in symptomatic PFP patients.

## 2. Materials and Methods

### 2.1. Participants

A convenience sample of ten participants (five males, five females) was recruited. Participants reviewed and signed an informed consent form before beginning the study. The participants completed a brief questionnaire asking about gender, age, and previous injury in the past six months. The protocols of this study were reviewed and approved by the Institutional Review Board of the affiliated university and all study activities followed the principles of the Declaration of Helsinki as revised in 2013. To meet the inclusion criteria, participants needed to run a minimum of ten miles per week on average and be between the ages of 18 and 60. Participants were excluded if they (1) experienced any symptoms of PFP, (2) had a history of lower extremity injury or surgery within the past six months, or (3) required orthotics or shoe inserts for running. Participants were also excluded if they were experiencing any uncontrolled cardiovascular pathology or were told by a physician that they should not exercise.

### 2.2. Procedure

**Figure 1:**
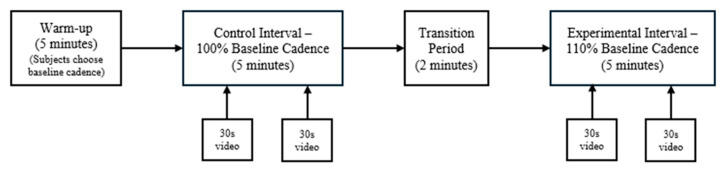
Protocol flowchart.

Instruction of the protocol was delivered, which consisted sequentially of a warm-up interval, followed by a control interval, a transition interval, and an experimental interval (Figure 1). To evaluate lower body kinematics, visual landmarks were drawn onto the bilateral lower extremities of the participants in preparation for post-performance video analysis: mid-posterior thigh, 25 cm inferior to the greater trochanter; mid-popliteal fossa along the tibiofemoral joint line; and mid-calf, 20 cm superior to the lateral malleolus [24].

During the warm-up interval, participants self-selected the treadmill speed at a moderate intensity that they could maintain for 20 min at most. This interval lasted five minutes, during which time the subject’s baseline cadence was established by counting the number of right foot strikes observed in 60 s and multiplying that by two.

After the warm-up interval, the participants transitioned to the control interval. During this interval, the participants ran for five minutes at 100% of the baseline cadence established during the warm-up. An audible metronome was used to ensure the participants maintained a steady pace. A 30 s video recording was taken, and heart rate was measured at the 2- and 4-min marks of the interval.

Once the control interval was completed, the participants entered a 2 min transition period to allow for adjustment to running at 110% of their baseline cadence. The speed of the treadmill was not changed; participants were only instructed to increase their cadence. The participants then entered the experimental phase, which consisted of running at 110% of their established cadence for 5 min. The metronome was used again to ensure participants adhered to the cadence. A 30 s video recording was taken, and heart rate was measured at the 2- and 4-min marks of the interval. Following this interval, the participants cooled down as needed.

### 2.3. Data Acquisition

Video recordings were taken using multiple iPhone 12 Pro (Apple Corp., Cupertino, CA, USA, 2020) devices with a phone tripod. The video recordings in the control and the experimental intervals were taken from the posterior view to capture the frontal plane and from the left side to capture the sagittal plane. The video recordings were synchronized to ensure the subject was in midstance (defined as the period in running gait when the center of mass is located directly over the support limb, and the knees are side by side) when establishing the amount of dynamic knee valgus [25]. The synchronized dual-camera recordings were then analyzed using the Dartfish mobile app (Dartfish Inc., 2020; The Gates at McGinnis Ferry, 4080 McGinnis Ferry Suite 1005, Alpharetta, GA 30005, USA), which is supported by research to evaluate frontal plane variables [25,26,27]. Using this two-dimensional analysis, average peak knee valgus angles were determined for both the right and left leg during the contact phase at midstance at the 2- and 4-min marks of each interval. These angles were established by the Dartfish mobile app, which measures the angle created by the midline of the femur and midline of the tibia using the previously drawn landmarks. The two measurements were averaged to determine the knee valgus angle during the control and experimental intervals.

### 2.4. Statistical Analysis

All data analyses were performed using IBM SPSS 28.0 (IBM Corp., Armonk, NY, USA) for Windows. An *a prior* alpha of 0.05 was selected. A paired sample, two-tailed *t*-test was used to determine the significance between knee angles obtained in the control and experimental intervals for each participant. A paired sample, one-tailed *t*-test was used to examine the difference in max heart rate (HR) during the control and experimental intervals. An independent samples *t*-test was conducted to compare baseline cadence, experimental cadence, and knee valgus angles of the left and right knees during the control and experimental phases, to assess gender differences. An initial sample size calculation estimated the required number of subjects to be *n* = 20. The study had difficulty recruiting participants, so the results were analyzed after 10 subjects. The results showed a statistically significant difference between the control and experimental phases. An after-study power analysis was performed as well. Cohen’s d was used as an estimated effect size. We determined an effect size of 0.70 on the R leg and 1.0 on the L leg. Being conservative, we chose the 0.70 effect size and calculated an achieved power of 0.88, well above the standard of 0.80 typically used.

## 3. Results

Ten runners (age = 26.9 ± 3.14 years old) were included in the study, with each runner representing both an experimental and control participant. No significant differences were observed between genders for any variable, suggesting the sample represented a homogeneous group. Participants’ preferred running speeds ranged from 2.4 to 3.8 m/s (2.81 ± 0.38 m/s) and preferred step rates (baseline) ranged from 150 to 176 (161.2 + 6.81) steps per minute. The step rate during the experimental cadence condition increased to 165–193 (176.9 + 7.43) steps per minute. During the control and experimental portions, average angles of the left leg were recorded as 4.46° ± 1.71° and 2.41° ± 1.13°, and average angles of the right leg were recorded as 4.57° ± 1.33° and 2.35° ± 1.06°, respectively. Combined, the average during the control was 4.52° ± 1.49° and 2.38° ± 1.07° for the experimental portion. The average change measured in the control versus experimental portions was a decrease of 2.23° and 2.05° in the amount of dynamic knee valgus in the right and left legs, respectively. A significant difference was found between the angles of each leg during the control and experimental phases, for both the left leg (*p* < 0.001) and the right leg (*p* < 0.001), as well as combined (*p* < 0.001) [Table 1]. As such, the results showed a significant decrease in the amount of dynamic knee valgus from running at a baseline cadence to running at a 10% increased cadence.

An analysis of measures from the control and experimental intervals was performed to determine consistency throughout the experiment. A strong positive correlation (0.768) was seen between maximal heart rates taken during control and experimental intervals. Maximum heart rate ranged from 129 to 185 (160 + 18.98) bpm for the control interval and 138 to 194 (169.1 + 19.21) bpm for the experimental interval. A paired sample, a one-tailed *t*-test (*p* = 0.027), indicated a significant increase in HR by solely increasing the cadence.

## 4. Discussion

This study attempted to determine whether a change in frontal plane kinematics could be elicited by increasing running cadence in healthy participants. Based on the reduced dynamic knee valgus angles found during the experimental interval, it was determined that increasing running cadence in healthy participants at a constant speed resulted in reduced dynamic knee valgus angles in midstance. Previous literature has highlighted that increased cadence results in improved measures of stride length correlates, decreased peak vertical ground reaction force, and decreased peak hip adduction during stance [15,18]. These improvements are in direct opposition to that of the biomechanics seen in individuals with knee injuries such as iliotibial band syndrome [28,29], tibial stress fractures [30], and most relevant to this study, patellofemoral pain (PFP) [31]. Our study may help explain the reduction in pain and dysfunction following a gait retraining program seen in de Souza Junior et. al., specifically focusing on increased cadence [16]. The dynamic lateral pull of the patella with an increased knee valgus angle is a known factor in PFP, so any change seen in the angle of pull for the patella can positively contribute to improved biomechanics.

Based on the current body of literature, it remains uncertain whether these findings met the criteria for the minimum detectable change (MDC). Previous literature has assessed the MDC at the knee when observed from the frontal plane in various dynamic lower extremity tasks and determined an MDC of 7.80–9.80° depending on the task [32]. Additionally, a similar analysis performed on the single-leg hop test yielded a MDC of 3.83° [33]. The established MDC for knee valgus in the frontal plane appears to be dependent on the task and, to the authors’ knowledge, there is no established MDC for analysis of participants when running. As such, the findings from this study, in conjunction with previous literature, support that an increased cadence is one way to improve running form and potentially minimize the risk of knee injury (with emphasis on PFP) by reducing associated biomechanical factors such as an increase in resultant dynamic knee valgus [21].

Previous studies have included measures of metabolic efficiency and running economy when adjusting cadence in the form of VO^2^ max and heart rate [34,35]. A recent study by Oeveren et al. best identified the relationship between cadence and heart rate by a parabolic curve with an optimal zone for individual runners designated at the trough of the curve [35]. As such, heart rate was measured to determine any resultant physiologic changes. An increase in the average maximal heart rate from the control to the experimental interval (9.1 bpm) was found to be significantly different. This is in disagreement with the study by Hafer et al., which found that there were no changes in measures of running efficiency at both the initial implementation of an increased cadence as well as during a 12-month follow-up after a cadence retraining protocol [18]. Increasing cadence while running on a treadmill can lead to an increase in heart rate [36]. This is because muscles are working harder and require more oxygen, which makes the heart pump faster to meet the increased demand.

The current literature notes the potential of two-dimensional frontal plane analysis correlating to that of three-dimensional analysis [25,27]; however, the frontal plane motion of the knee is also impacted by movement in the transverse plane at the hip [37]. A study using a similar two-dimensional video software during a single leg hop test in symptomatic PFP participants established inter-rater reliability of 0.792 and concurrent validity ICC values of 0.65–0.95 in the non-symptomatic leg [33]. Additionally, a systematic review with meta-analysis of frontal plane knee kinematics with squatting, landing, and cutting, found moderate reliability and poor validity using two-dimensional analysis, with the authors stating the current evidence is unable to support this type of analysis under similar scenarios [38]. However, prior research by Maykut et al. using the same software as this experiment, Dartfish, yielded high intra-rater reliability (ICCs: 0.955–0.976) for bilateral peak knee valgus in running participants [26]. The determination of concurrent validity by assessing the relationship between two-dimensional and three-dimensional measures yielded inconsistent results; however, it seems like validity may be task dependent.

This study is not without limitations. Even though previous research supports the reliability of 2-dimensional video analysis in running, results should be confirmed using a 3-dimensional system as well. This study also used only healthy asymptomatic runners without PFP, so it should be extrapolated to symptomatic patients with caution. Although the study was performed with limitations, including a lack of longitudinal analysis and participants limited to running less than three miles in total, the significance of these findings when compared with existing literature is promising, warranting the need for future research. Future research should continue to explore the long-term effects of cadence manipulation on symptomatic individuals and investigate the optimal cadence adjustments for different populations. Additionally, combining cadence retraining with other interventions, such as strength training for the hip abductors and external rotators, may further enhance the effectiveness of these programs in preventing and managing PFP.

Increasing cadence in healthy participants as a form of running gait retraining improves biomechanical measures. Changes in running gait, specifically cadence, have been found to affect foot strike patterns [39], which can lessen vertical force throughout the leg [40], leading to a decrease in the potential for developing PFP symptoms [16]. These findings support other studies that have developed gait retraining protocols to improve running biomechanics and reduce the risk of injury [16,18,41]. Additionally, these findings also support published recommendations for clinic-based gait-retraining and interventions to improve lower extremity kinematics and injury-risk mitigation [42]. To achieve a meaningful reduction in knee valgus angles, these modifications require sustained implementation over time to facilitate fundamental changes in gait patterns. Consistent application is necessary to maintain the benefits associated with reduced knee valgus.

## 5. Conclusions

Our study found that increasing running cadence was associated with a statistically significant reduction in knee valgus angles during the midstance of the running phase. These results suggest that cadence manipulation may serve as an effective intervention for clinicians and coaches to optimize knee biomechanics and potentially reduce the risk of patellofemoral pain (PFP) in runners. Integrating cadence adjustments into training programs could enhance lower limb alignment, thereby decreasing stress on the patellofemoral joint. This biomechanical adjustment may help mitigate the onset of PFP and related knee injuries.

## Figures and Tables

**Table 1:** Paired samples test: *t*-test for equality of means.

	df	T-Value	95% CI	Difference in Knee Angle Control vs. Experimental Intervals
Right Knee Valgus	9	10.13	2.72–1.73	2.23
Left Knee Valgus	9	6.49	2.74–1.34	2.05
Combined Knee Valgus	19	11.35	2.53–1.74	2.14

All *p* scores were <0.05, thus all measurements were statistically significant.

## Data Availability

The data presented in this study are available on request from the corresponding author without undue reservation.
